# Adiponectin as a Potential Biomarker for Pregnancy Disorders

**DOI:** 10.3390/ijms22031326

**Published:** 2021-01-29

**Authors:** Carmen Pheiffer, Stephanie Dias, Babalwa Jack, Nompumelelo Malaza, Sumaiya Adam

**Affiliations:** 1 Biomedical Research and Innovation Platform (BRIP), South African Medical Research Council, P.O. Box 19070, Tygerberg, Cape Town 7505, South Africa; stephanie.dias@mrc.ac.za (S.D.); babalwa.jack@mrc.ac.za (B.J.); u12174506@tuks.co.za (N.M.); 2 Division of Medical Physiology, Faculty of Health Sciences, Stellenbosch University, P.O. Box 19063, Tygerberg, Cape Town 7505, South Africa; 3 Department of Reproductive Biology, University of Pretoria, Private Bag X169, Pretoria 0001, South Africa; 4 Department of Obstetrics and Gynaecology, University of Pretoria, Private Bag X169, Pretoria 0001, South Africa; sumaiya.adam@up.ac.za

**Keywords:** adiponectin, biomarker, pregnancy, gestational diabetes, preeclampsia, preterm birth, foetal growth

## Abstract

Adiponectin is an adipocyte-derived hormone that plays a critical role in energy homeostasis, mainly attributed to its insulin-sensitizing properties. Accumulating studies have reported that adiponectin concentrations are decreased during metabolic diseases, such as obesity and type 2 diabetes, with an emerging body of evidence providing support for its use as a biomarker for pregnancy complications. The identification of maternal factors that could predict the outcome of compromised pregnancies could act as valuable tools that allow the early recognition of high-risk pregnancies, facilitating close follow-up and prevention of pregnancy complications in mother and child. In this review we consider the role of adiponectin as a potential biomarker of disorders associated with pregnancy. We discuss common disorders associated with pregnancy (gestational diabetes mellitus, preeclampsia, preterm birth and abnormal intrauterine growth) and highlight studies that have investigated the potential of adiponectin to serve as biomarkers for these disorders. We conclude the review by recommending strategies to consider for future research.

## 1. Introduction

Pregnancy is a unique physiologic state that is associated with several physiological, biochemical and metabolic adaptations to ensure adequate delivery of nutrition to the developing foetus [1]. One of the most common metabolic adaptations during pregnancy is a progressive, temporary increase in insulin resistance, which peaks in the third trimester when foetal energy demands are the highest [2]. Insulin resistance has wide-ranging effects on physiological processes and has been shown to stimulate hepatic gluconeogenesis, reduce glucose uptake in skeletal muscle and adipose tissue, and increase lipolysis in adipose tissue [3]. Collectively, these processes allow fats to be broken down to meet the energy requirements of the mother, while glucose is shunted to the developing foetus to promote development [2,4].

The mechanisms that underlie pregnancy-induced insulin resistance are not completely understood, but is postulated to be due to changes in the hormonal milieu, with various tissues including adipose tissue, the placenta and ovaries thought to contribute to the state of insulin resistance [5]. Placental hormones such as human placental lactogen and placental growth hormone upregulate the growth hormone/insulin like growth factor axis to promote nutrient transfer to the foetus and enable foetal growth, while oestrogen, progesterone and adipocyte-derived hormones, such as adiponectin, leptin, resistin, tumour necrosis factor alpha, interleukin-6 and C-reactive protein, are also suggested to play a role in the development of insulin resistance during pregnancy [2,4,5,6,7]. In this context, the role of the adipose-derived hormone adiponectin is of interest, especially as this adipokine is implicated in a broad range of physiologic processes. Adiponectin is the major adipokine secreted mainly from adipose tissue and has been suggested to act as an insulin sensitizer that plays an important role in regulating insulin action and glucose homeostasis. In addition, adiponectin has pleiotropic effects on the regulation of energy homeostasis, systemic inflammation, vascular function, cell growth and bone metabolism. Accordingly, adiponectin concentrations are decreased during pregnancy, as well as obesity and obesity-related complications, such as type 2 diabetes, cardiovascular diseases and non-alcoholic fatty liver disease [4,5]. Much is known about the roles of adiponectin in regulating metabolic homeostasis, however, the role of this hormone during pregnancy remains to be fully elucidated.

In recent years, the identification of maternal factors that could predict the outcome of metabolically compromised pregnancies has attracted considerable interest. Such biomarkers could act as valuable tools that allow the early recognition of high-risk pregnancies, facilitating close follow-up and prevention of pregnancy complications in mother and offspring [8,9]. A variety of genetic, epigenetic and serum biomarkers have been explored as potential biomarkers of common pregnancy disorders [8,9]. However, despite extensive research in this field, none of the investigated biomarkers have yet achieved clinical applicability. In this review we consider the role of adiponectin as a potential biomarker of disorders associated with pregnancy. We first highlight the importance of identifying pregnancy biomarkers and, thereafter, discuss adiponectin within the context of common disorders associated with pregnancy (gestational diabetes mellitus (GDM), preeclampsia, preterm birth (PTB) and abnormal intrauterine growth (foetal growth restriction (FGR) and large for gestational age (LGA)). We conclude the review by recommending strategies to consider for future research.

## 2. Clinical Biomarkers for Pregnancy Outcomes

Investment in maternal health offers tremendous potential towards achieving the Sustainable Development Goal targets 3.1 (reducing the global maternal mortality ratio to less than 70 per 100,000 live births), 3.2 (ending preventable deaths of newborns and children under five years of age) and target 3.4 (reducing premature mortality from non-communicable diseases (NCDs) by a third). Pregnancy complications such as GDM, preeclampsia, PTB, FGR and LGA are associated with perinatal complications such as caesarean deliveries, birth trauma, macrosomia, hypoglycaemia, shoulder dystocia and respiratory distress syndrome, but importantly also create a predisposition to develop NCDs, such as obesity, type 2 diabetes, hypertension, and cardiovascular disease later in life [10]. Poor pregnancy health and an unfavourable intrauterine environment during critical developmental stages leads to a vicious cycle of intergenerational risk transmission through abnormal developmental programming in utero [11]. Thus, interventions during pregnancy offer an ideal opportunity to not only improve perinatal outcomes, but also to fight the burgeoning NCD epidemic that is ravaging health systems globally [10]. The NCD burden is a significant public health crisis as recently highlighted by the COVID-19 pandemic where a positive relationship between COVID-19 severity and NCD comorbidities has been reported [12].

**Figure 1:**
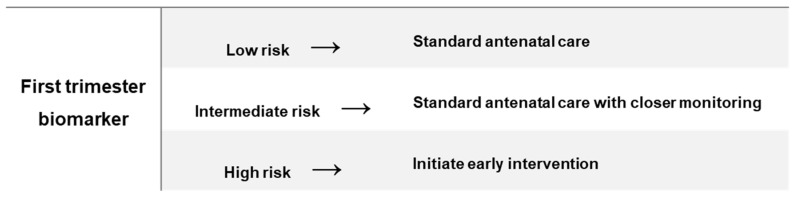
Clinical role of an early biomarker to prevent adverse pregnancy outcomes.

Early detection of pregnancy-related disorders is a major challenge that not only affect pregnancy health in low- and middle-income countries, but shortfalls of the currently available screening or diagnostic methods contribute to delayed detection and management of high-risk pregnancies in high-income countries [13]. The identification of specific biomarkers generated in the early stages of pregnancy offer tremendous potential to diagnose high-risk pregnancies and prompt the initiation of early preventive or therapeutic care, which may normalise homeostasis to prevent adverse pregnancy outcomes (Figure 1). Due to the inaccessibility of gestational tissues, molecules from maternal blood that are altered during the progression of pregnancy and are associated with the development of complications are a likely source of biomarkers. A range of maternal molecules, which include, proteins, metabolites, genetic and epigenetic factors have been widely explored as a source of pregnancy-related biomarkers, with varying levels of success [8,9]. Although highly plausible, it remains to be conclusively established whether interventions during pregnancy will prevent or reduce long-term maternal risks. Nonetheless, the development of rapid, cost-effective, point-of-care tests capable of identifying high-risk pregnancies offers opportunities for early preventative action. As such, biomarker discovery has become a major priority and has attracted increased interested in pregnancy research. Unfortunately, despite extensive research efforts, none of the investigated biomarkers have yet achieved clinical applicability [8,13]. Large scale, prospective population-based studies that are conducted across diverse environments and populations are required to advance biomarker discovery.

## 3. Adiponectin

**Figure 2:**
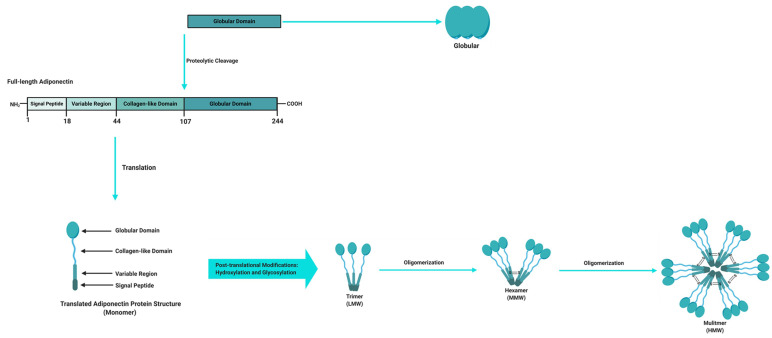
Adiponectin domains and molecular structure. The full-length structure of adiponectin is composed of a 244 amino acid protein mainly synthetized by adipocytes as a single subunit containing an N-terminal signal peptide, a variable region, a collagen-like domain and a globular domain at the C-terminus. Before it is secreted to the circulation, adiponectin undergoes oligomerization to form trimers (low molecular weight (LMW)), hexamers (medium molecular weight (MMW)), and multimers (high molecular weight (HMW)). Adiponectin is also present in plasma as a globular isoform synthesized by the proteolytic cleavage of the globular domain of the full-length adiponectin protein. Figure adapted and modified [20,22], and created with BioRender.com.

Adipose tissue is a powerful endocrine organ that secretes a variety of adipokines with important roles in the regulation of metabolic homeostasis. Adipokines that are produced and secreted from adipose tissue include adiponectin, leptin, TNFα and resistin, with the former being the most abundantly secreted adipokine and implicated as a key regulator of metabolic and inflammatory processes [14,15]. Adiponectin, also known as an adipocyte complement-related protein of 30 kDa (Acrp30), was first discovered in 1995 [16], while three other independent research groups also identified adiponectin as AdipoQ [17], adipose most abundant gene transcript 1 (apM1) [18] and gelatin-binding protein of 28 kDa (GBP-28) [19] using different approaches. Human adiponectin is a 244 amino acid protein that consists of an amino (NH2)-terminal signal sequence, a variable region, a collagenous domain and a carboxy (COOH)-terminal globular domain (Figure 2) [16,20,21]. Adipocytes secrete adiponectin into the bloodstream as three oligomeric complexes, including a low-molecular-weight (LMW) trimer (~60 kDa), a medium-molecular-weight (MMW) hexamer (~150 kDa), and a high-molecular-weight (HMW) multimer (~420 kDa), which are formed via a series of complex signalling and modification processes [20,21]. In its simplest form, adiponectin is a trimer, consisting of three monomers (~28 kDa) that undergo post-translational modifications (hydroxylation and subsequent glycosylation), and is stabilized by disulphide bonds within the collagenous domains of each monomer [20,22]. The trimers further multimerize via disulphide bonds at their collagenous domains to form hexamers and multimers, which are comprised of 12–32 monomers [20,22]. Globular adiponectin, which is synthesized by proteolytic cleavage of full-length adiponectin at the globular domain is also present at low levels in human plasma and is biologically active [21,22,23], while the monomeric form of adiponectin is undetected in the circulation and is restricted to adipocytes [24,25].

Adiponectin circulates at very high concentrations in human plasma, usually in a range of 2–30 µg/mL, which constitutes approximately 0.01% of total serum proteins [26,27,28]. HMW adiponectin is the predominant isoform in circulation and has been identified as the most active biological isoform, particularly in response to insulin sensitivity [24,29,30]. Sexual dimorphism in adiponectin regulation is observed, with higher levels of HMW expressed in women compared to men [31]. The injection of adiponectin deficient mice with HMW adiponectin reduced blood glucose concentrations [30], while mutations in the adiponectin gene that interfere with the formation of HMW adiponectin are associated with insulin resistance and type 2 diabetes [24]. As such, the ratio of HMW to total adiponectin, referred to as the adiponectin sensitivity index (SA), has been shown to be a better marker of insulin sensitivity, obesity and other metabolic disorders compared to total adiponectin [31]. Db/db mice, an obese diabetic mouse model, exhibit a reduced SA compared to db/+ control mice, while treatment with rosiglitazone increased the SA ratio in db/db mice [30]. Furthermore, Pajvani et al. showed that troglitazone treatment was associated with a higher SA index and improved insulin sensitivity and lowered hepatic glucose production in diabetic subjects and women with GDM [30]. Gastric bypass surgery is associated with an increased SA, which correlates with improved insulin sensitivity [32].

**Figure 3:**
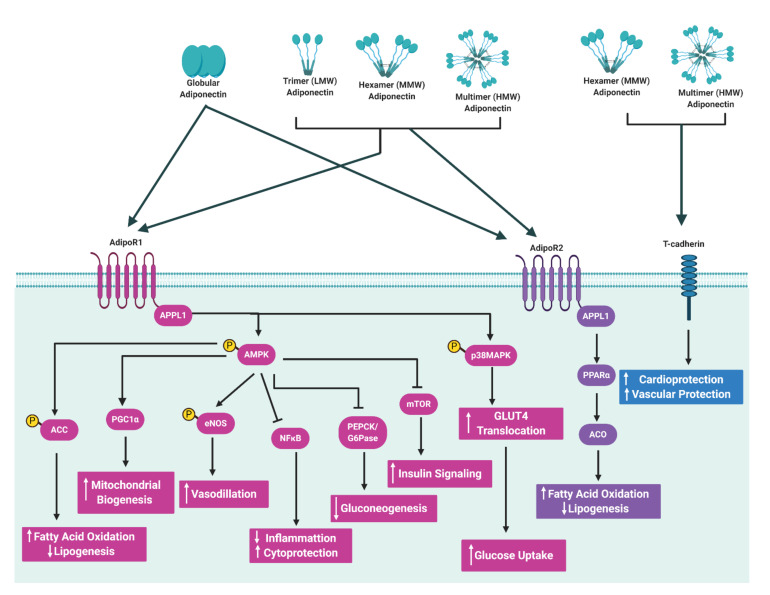
Schematic illustration of adiponectin intracellular signaling pathways. Adiponectin binds to its receptors (AdipoR1 and AdipoR2) and interacts with the adaptor protein containing a pleckstrin homology domain 1 (APPL1), leading to the activation of various signalling pathways including the 5′ adenosine monophosphate-activated protein kinase (AMPK) and peroxisome proliferator-activated receptor alpha (PPARα) pathways. Activation of these pathways lead to cellular responses such as the suppression of glucose production, lipogenesis and inflammation, and the stimulation of glucose uptake, fatty acid oxidation, mitochondrial biogenesis and increased insulin sensitivity. Black arrowheads indicate activation and flat lines indicate inhibition. White arrows pointing upwards represent an increase, while arrows pointing downwards represent a decrease in cellular processes. Illustration created with BioRender.com. Abbreviations: ACC, acetyl CoA carboxylase; ACO, acyl CoA oxidase AdipoR1, adiponectin receptor 1; AdpoR2, adiponectin receptor 2; AMPK, 5′ adenosine monophosphate-activated protein kinase; APPL1, adaptor protein containing a pleckstrin homology domain; eNOS, endothelial nitric oxide synthase, G6Pase, glucose 6-phosphatase; Glut4, glucose transporter 4; HMW, high molecular weight; LMW, low molecular weight; MMW, middle molecular weight; mTOR, mechanistic target of rapamycin; NFκB, nuclear factor kappa-light-chain-enhancer of activated B cells; p38MAPK, mitogen activated protein kinase; PEPCK, phosphoenolpyruvate carboxykinase, PGC1α, peroxisome proliferator activated receptor gamma coactivator 1 alpha; PPARα, peroxisome proliferator activated receptor alpha.

Although mainly produced and secreted by adipocytes, studies have provided evidence that adiponectin may be expressed at low levels in osteoblasts [33], liver parenchyma cells [34], myocytes [35] and possibly in placental tissue [36]. The biological actions of adiponectin are initiated when it binds and interacts with its receptors leading to the activation of downstream signaling pathways (Figure 3) [20,22]. Adiponectin receptor 1 (AdipoR1) and adiponectin receptor 2 (AdipoR2) are the main adiponectin receptors. AdipoR1 is ubiquitously expressed but occurs most abundantly in the skeletal muscle and has high affinity for globular adiponectin and low affinity for full-length adiponectin (LMW, MMW and HMW). AdipoR2 is predominantly expressed in the liver and has intermediate affinity for both globular and full-length adiponectin [37]. Other receptors such as T-cadherin are also capable of binding adiponectin and act as receptors for HMW and MMW isoforms in specific tissues such as muscle [38]. Upon adiponectin binding to its receptors, the adaptor protein containing a pleckstrin homology domain 1 (APPL1) protein binds to the intracellular domains of AdipoR1 or AdipoR2, thereby initiating a complex signal transduction cascade, which includes the activation of peroxisome proliferator-activated receptor-alpha (PPAR-α) and phosphorylation of AMP-activated protein kinase (AMPK) in peripheral tissues [20,22]. This leads to fatty acid oxidation and glucose uptake in skeletal muscle, and suppression of glucose production, lipogenesis and inflammation in the liver, ultimately improving insulin sensitivity [20,22,39]. Accordingly, low concentrations of adiponectin have been reported in common metabolic complication such as obesity and type 2 diabetes. Intriguingly, patients with type 1 diabetes have high serum adiponectin concentrations [40] and paradoxically high adiponectin concentrations have been shown to predict all-cause and cardiovascular mortality [41]. The reasons for these counterintuitive associations are unclear, although it has been speculated that high adiponectin concentrations may be due to counterregulatory mechanisms and adiponectin resistance.

**Figure 4:**
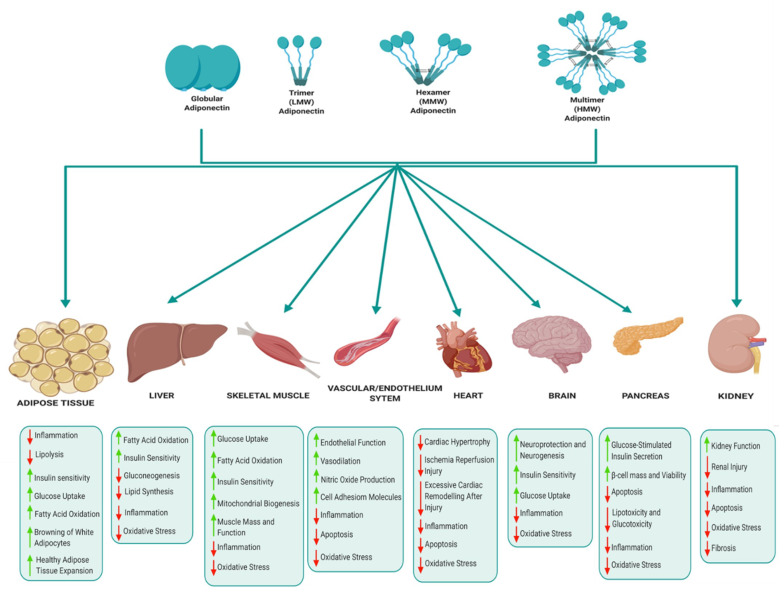
Schematic illustration of the pleiotropic effects of adiponectin. This figure provides a summary of the beneficial effects of adiponectin in key metabolic tissues including adipose tissue, liver, skeletal muscle, vascular/endothelium system, heart, brain, pancreas and kidney. Green arrows represent an increase, while red arrows represent a decrease. Illustration created with BioRender.com.

The various biological functions of adiponectin in key metabolic tissues are illustrated in Figure 4. In adipose tissue, adiponectin mediates anti-inflammatory effects by suppressing the expression of pro-inflammatory cytokines, improves lipid metabolism, glucose homeostasis and insulin sensitivity, and also promotes healthy adipose tissue expansion and browning of white adipocytes [42,43]. Adiponectin suppresses hepatic gluconeogenesis and improves glucose metabolism, insulin sensitivity, fatty acid oxidation and suppresses lipid synthesis, oxidative stress and inflammation in the liver [39,42,43]. In skeletal muscle, adiponectin regulates muscle mass and function, improves glucose metabolism, insulin sensitivity, mitochondrial biogenesis, fatty acid oxidation and reduces inflammation and oxidative stress [44,45]. Adiponectin also mediates cardioprotective actions and prevents atherosclerosis by improving endothelial function, prevents excessive cardiac remodelling following injury, suppresses cardiac hypertrophy and protects against ischemia reperfusion injury [43,46]. The cardioprotective effects of adiponectin are attributed to its anti-apoptotic, anti-oxidant and anti-inflammatory properties [46]. Adiponectin regulates appetite and energy expenditure in the hypothalamus, thus playing a central role in regulating energy homeostasis [47,48]. Adiponectin also improves insulin signalling and glucose homeostasis, alleviates inflammation and oxidative stress, and enhances neuroprotection and neurogenesis [49,50]. In the pancreas, adiponectin promotes glucose-stimulated insulin secretion by β-cells, protects β-cells by reducing apoptosis and promoting β-cells survival and viability [42,51]. Finally, adiponectin improves kidney function and protects against renal injury by reducing inflammation, oxidative stress, fibrosis and inhibiting apoptosis [52].

## 4. Adiponectin in Pregnancy

Adiponectin plays a critical role in gestational metabolic adaptations and regulating homeostasis during pregnancy [53]. Adipose tissue has been identified as the main source of circulating adiponectin during pregnancy [54]. It is still unclear to what extent adiponectin is expressed by the placenta [36,55,56]. Circulating adiponectin concentrations, primarily the HMW isoform, decrease during pregnancy, reaching its lowest levels in the third trimester when maternal insulin resistance is greatest [54,57,58]. An abundance of evidence demonstrates that low circulating adiponectin concentrations are associated with complications of pregnancy such as GDM, preeclampsia and an increased risk of delivering LGA or macrosomic infants, while hypoadiponectinemia during gestation and/or post-partum may predict future development of obesity and type 2 diabetes [55,59,60]. Although several lines of evidence support decreased adiponectin concentrations during pregnancy and pregnancy complications, conflicting results have been reported [55,59,61]. The reasons underlying these discrepant findings may be due to study design and population characteristics, but a possible explanation may be that increased adiponectin expression during gestation is a compensatory mechanism in response to severe insulin resistance. The adiponectin gene is regulated by DNA methylation, an epigenetic factor that is influenced by both genetic and environmental factors [60], which may add further complexity to adiponectin regulation during pregnancy. Moreover, adiponectin concentrations during pregnancy are affected by ethnicity and body mass index [58]. A longitudinal study of 80 pregnant women, demonstrated, as expected, progressively lower adiponectin concentrations with increasing gestation, but in addition, showed that adiponectin concentrations were lower in women of black ethnicity compared to white women, and in obese compared to normal-weight pregnant women, confirming previous findings in obese pregnant women [57]. These findings confirm racial disparities in metabolic risk, which may be due to fat distribution and insulin resistance [62], thus highlighting the importance of considering population characteristics in biomarker discovery. Adipokines function in a complex system, thus longitudinal assessment of various adipokines, such as leptin, are needed to understand the trajectories and dynamic associations of adipokines during pregnancy.

In addition to its potential to serve as a biomarker of poor pregnancy outcomes, studies have reported on the therapeutic potential of adiponectin. Using obese mouse models, studies demonstrated that normalizing adiponectin levels in obese pregnant mice in early pregnancy is able to significantly reduce the effects of maternal obesity on placental dysfunction and foetal overgrowth [63], and prevents adverse metabolic outcomes [64] and cardiac dysfunction [65] in offspring. In addition, adiponectin supplementation in dams with polycystic ovary syndrome was able to ameliorate the adverse effects of maternal high androgen levels on the metabolic health of adult female offspring [66]. Interestingly, dietary bioactive compounds such as polyphenols offer tremendous potential as cost-effective interventions to improve pregnancy complications [67]. Polyphenols are naturally occurring phytochemicals and secondary metabolites found in fruit, vegetables, cereals, nuts, tea, wine, chocolate, olives, spices and algae, that are widely reported to induce adiponectin levels and improve metabolic disorders such as obesity, type 2 diabetes and cardiovascular disease [67,68].

## 5. Adiponectin in Common Pregnancy Disorders

### 5.1. Gestational Diabetes

GDM is defined as glucose intolerance that develops during pregnancy and is the most common metabolic disorder during pregnancy [69]. The prevalence of GDM has steadily increased over the last 20 years, paralleling the rising obesity epidemic. Globally, it is estimated that 14% of pregnancies are affected by GDM [70], although rates vary between 1–28% depending on the population studied and the diagnostic criteria used [71]. The economic costs of GDM are high [72] and it has been estimated that the health care costs of treating women with GDM are ~25.1% higher than treating women without GDM [73]. The pathophysiology of GDM has not yet been fully elucidated, although several lines of evidence suggest that GDM occurs in pregnant women in whom β-cell function is not able to counteract insulin resistance that develops during pregnancy [74]. Insulin sensitivity is decreased by up to 40% in pregnant women with GDM compared to women with normoglycemia [75,76]. GDM is associated with an increased risk of perinatal complications and the development of future metabolic disease in both mother and child [77,78,79]. Early detection and treatment may reduce adverse pregnancy outcomes including stillbirth, neonatal macrosomia, neonatal hypoglycaemia, birth trauma and neonatal respiratory distress syndrome as well as decrease the risk of preeclampsia in the mother. The common risk factors, which include overweight or obesity, excessive gestational weight gain, advanced maternal age, family history of diabetes, previous history of GDM and adverse pregnancy outcomes, however, have poor predictive value and fail to identify a large percentage of women at risk for GDM, thus limiting their use as screening tools [71,80,81]. Currently the gold-standard for the diagnosis of GDM is the oral glucose tolerance test (OGTT) performed at 24–28 weeks gestation, although technical challenges, which include the requirement for fasting and multiple blood draws, and the high costs associated with sending blood samples to reference laboratories for testing, limit its widespread use [13]. The identification of biomarkers to facilitate the early detection of GDM, which may prompt early intervention to normalise blood glucose concentrations and prevent adverse pregnancy outcomes, is therefore a public health priority.

**Figure 5:**
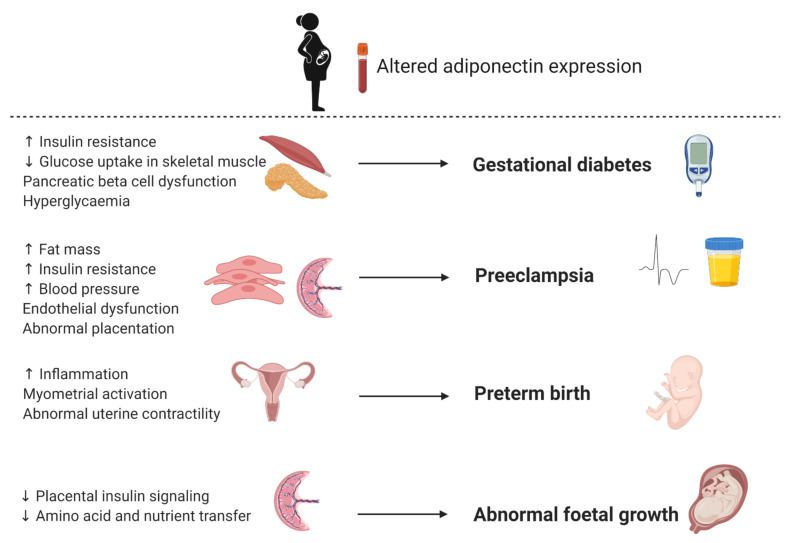
Schematic illustration of potential mechanisms linking altered adiponectin expression to pregnancy complications. Illustration created with BioRender.com.

In recent years, the identification of biomarkers for GDM has attracted considerable interest, with a number of genetic, epigenetic and serum markers identified as candidates [8]. One of these potential biomarkers is adiponectin, an anti-inflammatory adipokine with insulin sensitizing properties [20,22]. Decreased expression of adiponectin during pregnancy is postulated to augment insulin resistance in skeletal muscle, leading to decreased glucose uptake, pancreatic beta cell dysfunction, hyperglycemia and the development of GDM (Figure 5) [53]. As such, adiponectin has been extensively studied as a biomarker for GDM. A meta-analysis of 15 studies comprising 560 GDM patients and 781 controls demonstrated that maternal adiponectin concentrations are significantly lower in women with GDM compared to controls [82]. Furthermore, a longitudinal assessment of 445 [83] and 2590 [84] pregnant women showed that low adiponectin concentrations in the first trimester are associated with an increased risk of developing GDM during the second trimester. Recently, a meta-analysis that synthesized data from 11 studies comprising 2865 pregnant women showed that circulating adiponectin had a pooled odds ratio of 6.4 (95% CI 4.1, 9.9), a sensitivity of 64.7% (95% CI 51.0%, 76.4%) and a specificity of 77.8% (95% CI 66.4%, 86.1%) for predicting future GDM. Restricting studies to those that investigated first trimester adiponectin levels only, improved specificity (81.3% (95% CI 71.6%, 88.3%)), had no effect on the odds ratio (6.6 (95% CI 3.6, 12.1)), but decreased sensitivity (60.3% (95% CI 46.0%, 73.1%)) [85]. These results confirm that measurement of circulating adiponectin may improve the detection of women at high risk of developing GDM. However, conflicting findings have been reported. Ebert et al. reported that adiponectin concentrations were lower in pregnant compared to non-pregnant women but was not associated with GDM [86]. It should be noted that this small cross-sectional study consisted of 222 women only (74 per group), thus may have been underpowered to detect significant differences. Discrepancies between studies may be due to different diagnostic criteria and population characteristics. High inter-subject variability in basal adiponectin concentrations have been reported and highlights the importance of considering population characteristics in biomarker discovery. Thus, although adiponectin shows promise, at this time the clinical potential of using adiponectin as a biomarker to predict GDM remains to be established.

### 5.2. Preeclampsia

Preeclampsia is characterized by hypertension, proteinuria, endothelial dysfunction, and inflammation [87], and is a serious pregnancy-specific disorder which usually develops after 20 weeks of gestation. It affects approximately 5–8% of pregnancies globally [88] and is a leading cause of maternal and foetal mortality and morbidity [89,90]. The pathophysiological mechanism underlying preeclampsia remains elusive, although, several lines of evidence suggest that aberrant placentation is associated with early onset preeclampsia (<34 weeks gestation), while women with predisposing cardiovascular or metabolic risk for endothelial dysfunction develop late onset preeclampsia (>34 weeks gestation) [87]. These two stages of the disease relate to its severity, with early onset preeclampsia associated with a 20-fold greater mortality rate compared to late-onset [88]. Preeclampsia is associated with an increased risk of adverse perinatal outcomes, including PTB, intrauterine growth restriction, placental abruption, foetal distress, and foetal death in utero [91]. In addition to the foetal risk during pregnancy, there is growing evidence that preeclampsia has long-term adverse effects on the mother and offspring [91]. Although preeclampsia resolves after delivery, interventions for the management and prevention of foetal complications of preeclampsia during pregnancy are limited. Thus, efficient biomarkers, therapeutic targets, or therapeutic agents for the management of preeclampsia are urgently required to improve adverse perinatal health outcomes.

Adiponectin has attracted considerable interest as a biomarker of preeclampsia, particularly due to evidence that adiponectin may be involved in complex metabolic mechanisms associated with implantation, early pregnancy and placentation [92,93]. Moreover, serum adiponectin levels are inversely correlated with obesity, insulin resistance and hypertension [4,5], conditions related with an increased risk of preeclampsia. Although the mechanisms that underlie preeclampsia are not completed elucidated, it is speculated that increased fat mass, insulin resistance and blood pressure are associated with endothelial dysfunction and abnormal placentation, leading to the development of preeclampsia (Figure 5). Several studies have explored an association between adiponectin levels and preeclampsia, although, with conflicting results. Ramsay et al. was the first to show that serum adiponectin levels in the third trimester is significantly increased in women with preeclampsia compared to controls [94]. Naruse et al. similarly reported that serum adiponectin levels were elevated in women with preeclampsia compared to controls, after correcting for haematocrit [61]. These results were confirmed by Lu et al. and Takemura et al. who similarly showed elevated total and HMW serum adiponectin concentrations, respectively, in women with preeclampsia compared to women with normal pregnancies [95,96]. Other investigators observed that women with preeclampsia exhibited significantly lower serum adiponectin levels compared to controls. D’Anna et al. reported first trimester hypoadiponectinemia in pregnancies subsequently complicated by preeclampsia, which was inversely correlated to insulin resistance [97]. Furthermore, the authors showed that serum adiponectin concentrations were significantly lower in the late-onset subgroup compared to the early-onset subgroup. Thagaard et al. reported that first trimester adiponectin concentrations were significantly lower in severely obese pregnant women who later developed preeclampsia [98]. Similarly, Hendler et al. reported that overweight and obese women with severe preeclampsia had lower third trimester adiponectin concentrations compared to normal weight women [99]. However, when obese and overweight women were excluded, an increase in adiponectin concentrations was observed in normal weight women with preeclampsia compared to healthy controls. Discrepancies between studies may be due to factors such as sample size, ethnicity, and the timing of sample collection for adiponectin analysis. Adiponectin plays a role in several metabolic processes and its expression has been shown to change as pregnancy progresses. Other metabolic factors such as BMI, insulin resistance, and the onset and severity of preeclampsia may also affect adiponectin levels. Moreover, adiponectin consists of different molecular weight isoforms, whose relative expression and regulation varies during pregnancy. More research is required to assess the clinical potential of using adiponectin as a biomarker for preeclampsia.

### 5.3. Preterm Birth

Preterm birth (PTB) is defined as birth before 37 completed weeks of gestation and affects about 11% of pregnancies worldwide, ranging from 4% to 18% depending on geographic region and level of income of a country [100]. Low- and middle-income countries account for approximately 90% of global preterm births. PTB is the leading cause of mortality for children younger than five years, accounting for about 1 million neonatal deaths annually. Preterm infants who survive often present with poor neurodevelopment and cognitive disabilities [101] and behavioural and emotional difficulties [102] compared to term infants. Furthermore, their care places an increased burden on the health system [103,104] and causes immense psychological and financial burden to their families [105]. Iatrogenic PTB, which refers to PTB initiated by an obstetric care provider to prevent maternal or foetal complications accounts for approximately 30–35% of cases, while spontaneous PTB (due to cervical dilation or premature rupture of membranes) accounts for approximately 65–70% of cases [106]. Although the exact cause of spontaneous PTB is not known, it has been speculated that infection and/or inflammation are the most common pathological pathways associated with PTB. Bacterial colonization activates pro-inflammatory signals leading to inflammation of choriodecidual cells and activation of the cytokine-prostaglandin cascade, resulting in uterine contractions [107]. Currently, there are several predictive tests for PTB, which include ultrasound (cervical length measurement) [108,109], markers of intrauterine inflammation and infections such as the C reactive protein and proinflammatory cytokines [110,111]), markers of extracellular matrix degeneration (fetal fibronectin concentrations) [109], human chorionic gonadotropin [112], phosphorylated insulin-like growth factor-binding protein (phIGFBP-1) [108] and placental alpha microglobulin-1 (PAMG-1) [113]. Currently, cervical length measurement is the main test used to predict PTB, however, this measurement has low predictive value requiring its use in conjunction with other tests.

There is a need for a simple, affordable, rapid and safe tests to accurately predict PTB. However, the clinical utility and predictive ability of these tests vary across populations and studies [114]. Two meta-analyses of 116 different biomarkers from 217 studies over a period of four decades [115] and 30 novel biomarkers investigated over ten years [116] concluded that none of the investigated biomarkers can be considered clinically useful to predict PTB. In recent years, biomarkers that have been actively investigated for PTB include epigenetics (DNA methylation, histone modifications and microRNAs) [117], novel ultrasound markers (uterine artery pulsatility index, placental strain ratio and anterior cervical angle), and cervicovaginal, amniotic fluid and serum markers such as adipokines [118]. Adipokines play an important role in maternal-foetal adaptions and as such their expression has been demonstrated to vary during pregnancy and pregnancy complications [53,54,55]. Of these, expression of adiponectin in maternal serum is of interest as a predictor of PTB. Lower maternal serum concentrations of total, HMW and MMW adiponectin have been observed during PTB [119] and have been suggested to activate pro-inflammatory cytokines which infiltrate the uterine compartments resulting in premature uterine contractions, thereby activating the myometrial and leading to preterm delivery (Figure 5) [120]. An association between low adiponectin concentrations and pregnancy complications such as PTB have also been demonstrated in obese women [121]. Mierzyński et al. similarly observed lower maternal serum adiponectin concentrations in women with PTB compared to controls [122], although, this association may have been confounded by GDM. Despite the biomarker potential displayed by adiponectin, its expression is affected by factors such as ethnicity and BMI, thus further studies are required to assess the clinical utility of using maternal serum adiponectin as a predictor for PTB.

### 5.4. Foetal Growth

Foetal growth depends on a complex interaction between the maternal, foetal and placental environments. Many factors including hormones, nutrition, maternal age, parity, placental size, as well as genetic factors play a critical role in regulating foetal development [123,124]. Maternal factors affect the intrauterine environment and placental weight, which are important determinants of foetal growth [125]. An adverse intrauterine environment causes impaired foetal growth which can result in foetal growth restriction (FGR) and large for gestational age (LGA). Aberrant foetal growth patterns are associated with perinatal complications including PTB, caesarean deliveries, cardiac dysfunction and stillbirth [126,127,128], and an increased risk of developing metabolic diseases such as hypertension, cardiovascular disease, impaired glucose tolerance and metabolic syndrome in adulthood [129,130]. Current methods available for antenatal identification of FGR and LGA have poor sensitivity [9], with a large proportion of women remaining undetected. Thus, there is an urgent need to identify biomarkers that can prevent or delay the onset of adverse health outcomes.

Given the role of adiponectin in metabolic adaptation during pregnancy, it has been postulated that maternal and foetal adiponectin play a role in foetal development and growth. Dysregulated adiponectin expression inhibits the insulin signalling pathway and amino acid transport in the placenta, which affects nutrient transfer to the foetus and subsequently leads to abnormal foetal growth (Figure 5). Indeed, lower adiponectin concentrations in cord blood of LGA newborns compared to appropriate for gestational age (AGA) newborns have been observed [131]. A prospective cohort study of 300 pregnant women reported that maternal blood adiponectin concentrations decreased during pregnancy and was lower in mothers who gave birth to LGA compared to AGA babies [132]. These differences were statistically significant between 22–24 weeks of gestation, suggesting that adiponectin could serve as potential early biomarkers of LGA. However, conflicting results have been reported. Yalinbas et al. showed that cord blood adiponectin concentrations were lower in small for gestational age (SGA) newborns compared to AGA and LGA groups [133], while other studies showed no difference in cord blood adiponectin levels between SGA and AGA newborns [134] and in first trimester maternal adiponectin concentrations between FGR and healthy controls [135]. Conflicting results between studies may be due to study design and timing of blood collection for adiponectin analysis and population characteristics. Further studies are needed to confirm the clinical applicability of adiponectin as a biomarker for foetal growth.

## 6. Clinical Implications of Adiponectin Screening

Biomarkers are defined as “indicators of normal biological processes that can be used to detect disease or other biological states of organisms” [136]. In recent years, there has been overwhelming interest in biomarker research to aid clinical decision making. First trimester adiponectin concentrations are associated with the development of GDM, preeclampsia, PTB and abnormal intrauterine growth, thus has the potential to predict these high-risk pregnancies, facilitating closer follow-up and prevention of pregnancy complications in mother and child. Adiponectin has several of the attributes required of a biomarker; it is associated with disease progression, easily measured in plasma or serum, and can be quantified using cost effective, reliable, and reproducible tests. However, the incorporation of potential biomarkers, such as adiponectin, into clinical guidelines and practice is fraught with challenges, which include experimental design, sample quality, analytical issues with measurement, and importantly, the high costs associated with clinical trials to assess efficacy [136,137]. Furthermore, ethnic-, BMI- and gestational variation in adiponectin concentrations during pregnancy have been reported and could affect its clinical applicability [57,58,138]. Thus, more translational research is required before adiponectin can be useful in clinical practice.

## 7. Conclusion and Future Considerations

Pregnancy complications are an increasing public health crisis that adversely affect pregnancy outcomes and increases the risk for developing future metabolic disease for both mother and child. The identification of specific biomarkers generated in the early stages of pregnancy offers tremendous potential to diagnose high-risk pregnancies and prompt the initiation of early preventive or therapeutic care to improve pregnancy outcomes. Several maternal factors have been investigated as potential biomarkers, with circulating adiponectin concentrations showing promise. However, studies to date have yielded conflicting findings pertaining to adiponectin in pregnancy, thus further investigation in this area is essential. Elucidation of adiponectin physiology in the setting of both normal pregnancy and its pathologic conditions may provide unique insight into fundamental processes that are relevant to health and disease in mother and child. This requires well-designed prospective studies with longitudinal assessment of adipokines during pregnancy to understand the trajectories and dynamic associations of adipokines during both normal and complicated pregnancies. Such studies should include the effectiveness of screening and treatment approaches based on first-trimester high-risk predictors, including quality of life and health indicators, and the economic costs of treating both short-and long-term adverse pregnancy outcomes. Importantly, it should be acknowledged that no single biomarker may be effective as a sensitive, specific, and robust test, and that a combination of adiponectin and maternal risk factors may be required for risk prediction algorithms.
